# Association between atmospheric particulate matter pollution during pregnancy and premature birth in China: a meta-analysis

**DOI:** 10.3389/fpubh.2025.1474134

**Published:** 2025-01-23

**Authors:** Bingbing Guo, Xinye Jiang

**Affiliations:** Wuxi Maternity and Child Health Care Hospital, Women's Hospital of Jiangnan University, Jiangnan University, Wuxi, Jiangsu, China

**Keywords:** pregnancy, atmospheric particulate matter, premature birth, meta-analysis, matter pollution

## Abstract

**Objective:**

The impact of maternal exposure to outdoor particulate matter during pregnancy on preterm birth is still inconsistent, particularly under the unique atmospheric particulate matter pollution conditions in China, where the effects on preterm birth remain poorly understood. The study intends to evaluate the correlation between atmospheric particulate matter pollution (PM2.5 and PM10) during pregnancy and premature birth in China through a Meta-analysis.

**Methods:**

The Chinese databases (CNKI and Wanfangdata), and the English databases (PubMed, Web of Science, and Embase) were searched to collect literature related to exposure to atmospheric particulate matter during pregnancy in China and premature birth. A Meta-analysis was conducted using Stata12.0 software.

**Results:**

A total of 29 studies were included in this study (15 cross-sectional studies, 11 cohort studies, and 3 case–control studies), covering more than 30 provinces (municipalities directly under the Central Government) in China, with a total sample size of 9,283,110 people. The Meta-analysis results showed that the risk of premature birth with the OR value was 1.03 (95%CI:1.011.06) for exposure to PM2.5 in mid-pregnancy, 1.03 (95%CI:1.011.04) for exposure to PM2.5 in late pregnancy, 1.07 (95%CI:1.051.10) for exposure to PM2.5 throughout pregnancy, and 1.04 (95%CI:1.001.07) for exposure to PM10 throughout pregnancy. No correlation was found between exposure to atmospheric particulate matter at other times and the occurrence of premature birth.

**Conclusion:**

Although our results indicate that exposure to atmospheric particulate matter during the second and third trimesters of pregnancy increases the risk of preterm birth among pregnant women in China, the association is relatively weak. Additionally, the results may be influenced by potential confounding factors. Therefore, further detailed research is needed to explore the relationship between particulate matter exposure and preterm birth or other adverse pregnancy outcomes.

## 1 Introduction

Public health problems caused by atmospheric particulate matter pollution (PM2.5 and PM10) have attracted attention from global health departments. Due to industrialization, the concentration of particulate matter in the air is generally higher in developing countries than in developed ones. This pollution can lead to adverse outcomes for pregnant women, particularly premature birth. Airborne particulate matter pollution has become a major risk factor for global disease (1), with fetuses being particularly vulnerable to toxic substances during pregnancy. Exposure to these airborne particulates can affect pregnant women, leading to adverse outcomes such as fetal growth restriction, low birth weight, preterm birth, and even stillbirth. In developing countries, one-fifth of low birth weight cases and one-quarter of stillbirths can be attributed to air pollution from the use of solid fuels (2). Studies have shown that particulate pollutants in the air can enter the placenta through simple diffusion, with the absorbed particles circulating throughout the body via the bloodstream, affecting organs and tissues, and causing metabolic disorders by inhibiting or disrupting certain enzyme activities, ultimately impairing fetal growth and development (3). In recent years, biological mechanisms behind preterm birth in pregnant women have been linked to inflammation, immune response, and endocrine regulation (4). However, the specific biological mechanisms by which prenatal exposure to particulate matter impacts preterm birth remain unclear. Some studies suggest that air pollutants may activate the fetal hypothalamic–pituitary–adrenal axis, triggering uterine contractions and premature rupture of membranes, leading to preterm birth (5). Other studies indicate that while air pollution may not directly cause maternal infections, exposure to pollutants can impair immune function, increasing maternal susceptibility to infections (6), which may, in turn, trigger early uterine contractions and result in preterm birth.

However, current research on the correlation between atmospheric particulate matter pollution and preterm birth is inconsistent. Some studies have found a significant association between air pollution exposure and an increased risk of preterm birth (7, 8), while other research has shown no correlation between particulate matter exposure at different stages of pregnancy and preterm birth, with some studies even suggesting that such exposure could act as a protective factor against preterm birth (9). Research in China on the correlation between air pollutant exposure and preterm birth also presents mixed results. This may be due to differences in geographical location, economic development status, pollutant concentrations, or the timing of exposure during pregnancy. Therefore, it is necessary to conduct research based on the specific conditions of China. This paper used a meta-analysis approach to analyze the correlation between exposure to particulate matter pollution at different stages of pregnancy and preterm birth among pregnant women in China, providing scientific evidence for further exploration of the impact of air pollutants on pregnancy outcomes.

## 2 Materials and methods

### 2.1 Sources of information and literature search

Using a combination of subject words and keywords, we searched for “particulate matter” or “air pollution,” “adverse birth outcomes” or “premature birth,” “pregnancy” or “gestation” in Chinese databases. In English databases, the MeSH terms and free words were used for search conditions, with “Adverse birth outcomes” OR “Premature birth” AND “Air pollution” OR “PM2.5” OR “PM10” OR “PM2.5” AND “Airborne particulate matter” AND “China” in PubMed, Web of Science, and Embase. The search covered the period from the establishment of the database from January 1, 2020 to April 30, 2023, collecting all research on the correlation between outdoor atmospheric particulate matter pollution and premature birth among pregnant women in China. The search was supplemented with a literature backtracking method to further search for literature data. The literature search was not restricted by language.

### 2.2 Literature screening and data extraction

Inclusion criteria: ① The study sample was from China and the investigation area was in China; ② The age of the research subjects was ≥18 years old and they did not suffer from other serious physical diseases; ③ The included studies could provide at least one OR between the exposure factor and premature birth and 95% confidence interval, or the data in the text could be converted to OR and 95% confidence interval. Exclusion criteria: ① Repeated publication or incomplete data information; ② The research design and statistical methods are unreasonable; ③ Reviews or expert comments, etc. According to the search strategy, the literature was searched, and two researchers screened the retrieved literature, and finally, one person extracted the data according to a unified data form, including the first author, investigation year, research area, research features, sample size, exposure period, exposure factors, etc. Premature birth in this paper is defined as giving birth between 28 and 37 weeks of pregnancy; PM2.5 refers to particles in the environment with a diameter less than or equal to 2.5 microns; PM10 refers to particles in the environment with a diameter less than 10 microns.

### 2.3 Literature quality evaluation

The Newcastle-Ottawa Scale (NOS) was used to evaluate the quality of the included literature. The scale includes eight items, including the selection of research objects, comparability, exposure or results. The score ranges from 0 to 9 points. The higher the score, the higher the quality of the literature.

### 2.4 Statistical analysis

Stata12.0 software was used to test the heterogeneity of the pooled data. If there was high heterogeneity (I2 ≥ 50%), a random effects model was adopted; If there was medium or low heterogeneity (I2 < 50%), a fixed effects model was used for analysis, subgroup analysis was conducted to explore the sources of heterogeneity. Sensitivity analysis was conducted by excluding any one of the articles and comparing the results. If the results changed little, the results were considered stable. Publication bias was evaluated by drawing a funnel plot and conducting an Egger test. All statistical tests were considered significant at *p* < 0.05.

## 3 Results

### 3.1 Basic characteristics

**Figure 1:**
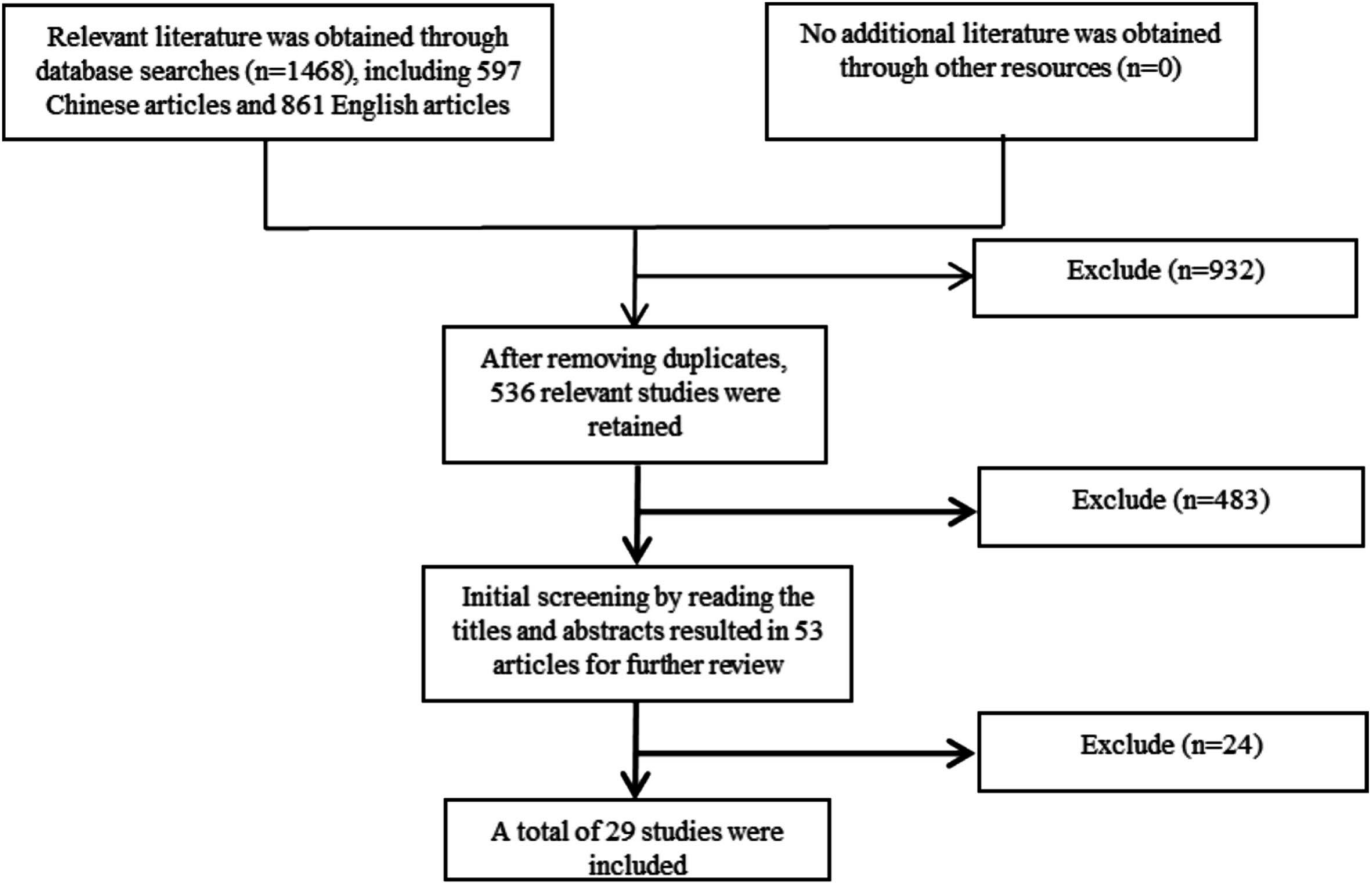
Literature search flowchart.

Overview of included studies: A total of 1,468 articles were initially retrieved (including 861 English articles). After screening according to the inclusion and exclusion criteria, 29 articles were finally included, including 20 in English and 9 in Chinese. The literature covered more than 30 provinces (municipalities directly under the central government) in China (Figure 1). The types of studies included 15 cross-sectional studies, 11 cohort studies, and 3 case–control studies. The total sample size of the studies included in this study was 9,283,110. In terms of literature quality, the average NOS score was 7.45 ± 0.69, with 26 articles scoring >7 points and 3 articles ≤6 points (Table 1).

**Table 1:** Basic information and quality scores of included literature.

References	Location	Period	Type	Sample size	Exposure period	Exposure factor	NOS
Zhang et al. (29)	Taiyuan	1997–2004	Cross-sectional study	52,951	Early, mid, and late pregnancy	PM10	7
Yakupu et al. (30)	Beijing	2007–2009	Case–control study	25,872	Early pregnancy	PM10	8
Zhao et al.(31)	Lanzhou	2010–2012	Cross-sectional study	8,969	Early, mid, late pregnancy, and entire pregnancy period	PM10	8
Qian et al. (32)	Wuhan	2011–2013	Cohort study	95,911	Entire pregnancy period	PM2.5, PM10	8
Huang et al. (33)	Beijing	2006–2010	Cross-sectional study	50,874	Early, mid, and late pregnancy	PM10	7
Yang et al. (34)	Shanghai	2013	Cross-sectional study	38,083	Early, mid, late pregnancy, and entire pregnancy period	PM10	7
Liu et al. (22)	Shanghai	2013	Cross-sectional study	195,400	Entire pregnancy period	PM2.5	8
Ye et al. (19)	Taizhou	2013–2016	Retrospective cohort	26,998	Early, mid, late pregnancy, and entire pregnancy period	PM2.5, PM10	7
Li et al. (20)	Beijing	2013–2014	Cohort study	1,280,524	Early, mid, late pregnancy, and entire pregnancy period	PM2.5, PM10	8
Wang et al. (35)	Guangdong	2015–2017	Retrospective cohort	506,280	Early, mid, late pregnancy, and entire pregnancy period	PM2.5, PM10	8
Guo et al. (36)	30 Provinces	2014	Cohort study	426,246	Early, mid, late pregnancy, and entire pregnancy period	PM2.5	7
Zhang (37)	Gansu	2010–2012	Cross-sectional study	10,542	Early, mid, late pregnancy, and entire pregnancy period	PM2.5, PM10	8
Liu et al. (38)	Guangdong	2014–2015	Case–control study	3,550	Early, mid, late pregnancy, and entire pregnancy period	PM2.5, PM10	8
Xiong et al. (39)	Changsha	2015–2017	Retrospective study	344,880	Entire pregnancy period	PM2.5, PM10	8
Liang et al. (40)	9 Cities	2014–2017	Cohort study	1,455,026	Early, mid, and late pregnancy	PM2.5	8
Ji et al. (41)	Shanghai	2014–2015	Cross-sectional study	25,493	Early, mid, late pregnancy, and entire pregnancy period	PM2.5	6
Liang (42)	7 Cities	2015–2017	Cross-sectional study	308,201	Early, mid, late pregnancy, and entire pregnancy period	PM2.5	6
Yuan et al. (43)	Shanghai	2013–2016	Cohort study	3,692	Early, mid, late pregnancy, and entire pregnancy period	PM2.5	7
Zhang (44)	Wuhan	2015	Cross-sectional study	2,101	Early, mid, late pregnancy, and entire pregnancy period	PM2.5	8
Yang (45)	Zhejiang	2013–2017	Cross-sectional study	6,274	Early, mid, and late pregnancy	PM2.5	7
He et al. (46)	336 Cities	2010–2015	Cohort study	3,723,169	Entire pregnancy period	PM2.5	7
Chu et al. (47)	8 Provinces	2009–2011	Cohort study	5,976	Early, mid, late pregnancy, and entire pregnancy period	PM2.5	6
Chen et al. (48)	Shiyan	2015–2017	Cross-sectional study	13,111	Early, mid, late pregnancy, and entire pregnancy period	PM2.5, PM10	8
Zhang et al. (49)	Henan	2013–2018	Cross-sectional study	196,780	Early, mid, late pregnancy, and entire pregnancy period	PM2.5	7
Su et al. (50)	Shanghai	2014–2020	Retrospective cohort study	179,385	Early, mid, and late pregnancy	PM2.5, PM10	8
He et al. (51)	Yanan	2018–2019	Cohort study	10,160	Early, mid, late pregnancy, and entire pregnancy period	PM2.5, PM10	7
Li et al. (23)	Henan	2015–2018	Cross-sectional study	275,380	Early, mid, late pregnancy, and entire pregnancy period	PM2.5, PM10	8
He et al. (52)	Shanghai	2016	Cross-sectional study	10,370	Early, mid, late pregnancy, and entire pregnancy period	PM2.5	8
He et al. (53)	Yanan	2018–2019	Case–control study	912	Entire pregnancy period	PM2.5, PM10	8

### 3.2 Meta-analysis result on the correlation between PM2.5 exposure during different pregnancy periods and premature birth

**Figure 2:**
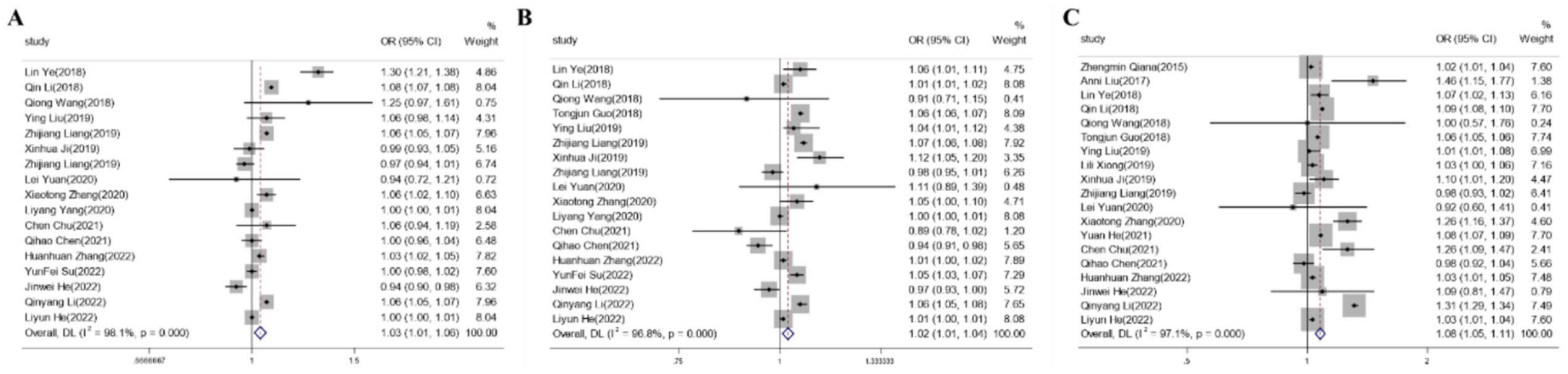
The Forest plot of correlation between PM2.5 exposure during different pregnancy periods and premature birth. **(A)** Mid-pregnancy exposure to PM2.5. **(B)** Late-pregnancy exposure to PM2.5. **(C)** Pregnancy-period exposure to PM2.5.

The meta-analysis results showed that the odds ratio (OR) for the risk of preterm birth due to PM2.5 exposure during the second trimester was 1.03 (95% CI: 1.011.06); for the third trimester, the OR was 1.03 (95% CI: 1.011.04); for the entire pregnancy, the OR was 1.07 (95% CI: 1.05 ~ 1.10) (Figure 2).

### 3.3 Meta-analysis result on the correlation between PM10 exposure during different pregnancy periods and premature birth

**Figure 3:**
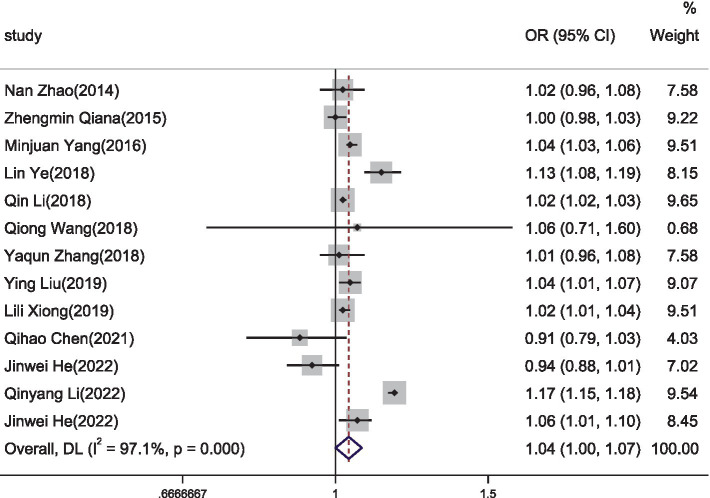
The forest plot of correlation between PM10 exposure during different pregnancy periods and premature birth.

This study found that for the entire pregnancy period, the OR for the risk of preterm birth due to PM10 exposure was 1.04 (95% CI: 1.00 ~ 1.07) (Figure 3).

### 3.4 Subgroup analysis

The meta-analysis results revealed significant heterogeneity among studies investigating the relationship between PM2.5 and PM10 exposure and preterm birth. We conducted a subgroup analysis to explore the sources of this heterogeneity. The analysis was performed based on different subgroups, including investigation year (before and after 2015), study type (cross-sectional, cohort, and retrospective studies), study region (southern, northern, or mixed regions), and sample size (whether the size was ≥100,000). In the meta-analysis of PM2.5 and preterm birth, the subgroup analysis found that study type explained part of the heterogeneity, while no heterogeneity sources were found in the other subgroups. In the meta-analysis of PM10 and preterm birth, the subgroup analysis did not identify any significant sources of heterogeneity (see Tables 2, 3).

**Table 2:** Subgroup analysis of the relationship between PM2.5 exposure and preterm birth.

Subgroup	Number of studies	Sample size	Heterogeneity I^2^ (%)	Test *P*-value	Pooled OR (95% CI)
Investigation year					
≤ 2015	11	5,983,739	90.5	<0.05	1.06 (1.04–1.08)
> 2015	8	1,470,483	98.5	0.127	1.09 (0.98–1.21)
Study type					
Cross-sectional	8	1,026,836	98.6	0.021	1.12 (1.02–1.23)
Cohort	7	5,545,678	92.1	<0.05	1.07 (1.04–1.09)
Retrospective	4	220,427	12.8	0.012	1.03 (1.01–1.05)
Geographic region					
Southern	12	1,535,987	76.5	0.002	1.04 (1.01–1.07)
Northern	4	1,762,844	99.2	0.033	1.13 (1.01–1.27)
Mixed	3	4,155,391	88.4	<0.05	1.07 (1.05–1.10)
Sample size					
≤ 100,000	10	197,362	77.4	0.001	1.05 (1.02–1.08)
> 100,000	9	7,256,860	98.4	<0.05	1.09 (1.05–1.13)

**Table 3:** Subgroup analysis of the relationship between PM10 exposure and preterm birth.

Subgroup	Number of studies	Sample size	Heterogeneity I^2^ (%)	Test *P*-value	Pooled OR (95% CI)
Investigation year					
≤ 2015	7	1,464,577	78.0	0.001	1.03 (1.01–1.05)
> 2015	6	1,150,723	97.7	0.534	1.03 (0.94–1.12)
Study type					
Cross-sectional	5	346,085	97.6	0.338	1.04 (0.961–1.12)
Cohort	3	1,386,595	73.8	0.891	1.00 (0.97–1.03)
Retrospective	5	882,620	77.4	0.003	1.06 (1.02–1.10)
Geographic region					
Southern	8	1,037,782	73.9	0.005	1.03 (1.01–1.06)
Northern	5	1,577,518	99.0	0.341	1.04 (0.96–1.13)
Mixed					
Sample size	9	208,296	76.2	0.040	1.03 (1.00–1.06)
≤ 100,000	4	2,407,064	99.2	0.122	1.07 (0.983–1.16)

### 3.5 Publication bias and sensitivity analysis

For studies with statistically significant differences in the combined Meta results, a funnel plot was drawn and symmetry was generally found. Egger’s test was used to test for publication bias, with t-values of 0.06, 0.08, 0.03, and 0.43, and *p*-values of 0.95, 0.94, 0.75, and 0.67, respectively, suggesting no publication bias. The sensitivity analysis of each combined result, after excluding any one of the studies, showed no significant changes in the overall results, suggesting good stability.

## 4 Discussion

Airborne particulate matter (PM2.5, PM10) consists of both anthropogenic and natural substances and is a mixture of various compounds, often accumulating heavy metals and toxic organic pollutants on its surface (10). The health issues caused by particulate matter pollution have gained widespread social attention, especially as pregnancy represents a vulnerable window period during which exposure to particulate matter can lead to fetal development issues or adverse outcomes such as preterm birth. Some studies have found that certain metal components of PM2.5, such as Ni and Pb, have been confirmed to have embryotoxic and teratogenic effects in various animal models, and they may also affect intrauterine growth in humans (11). Recent epidemiological studies have shown that PM2.5 exposure is associated with the incidence and development of adverse birth outcomes (12), including low birth weight (LBW), preterm birth (PTB), small for gestational age (SGA) (13), low birth length (14), and stillbirth (15). Factors contributing to adverse outcomes like preterm birth include genetic factors, environmental factors, or a combination of both. Although the etiology of preterm birth is highly complex, it is generally believed to be related to infections, inflammation, placental diseases, depression, anxiety, and immune factors (16). Exposure to high concentrations of particulate matter during pregnancy can impair the immune system of pregnant women, increasing the likelihood of preterm birth (17). Other studies have found that exposure to particulate matter during pregnancy can increase the number of cytotoxic cells in cord blood, leading to preterm birth (6). Although many studies confirm that exposure to particulate matter during pregnancy results in adverse birth outcomes such as preterm birth (18), there is still insufficient scientific evidence in China. Therefore, this study collects data on the correlation between exposure to pollutants such as PM2.5 and PM10 during different stages of pregnancy (early, mid, late, and the entire pregnancy) and preterm birth in pregnant women in China.

We compiled literature on the exposure of Chinese pregnant women to particulate pollutants (PM2.5 and PM10) at different stages of pregnancy and found a correlation between exposure to PM2.5 during the second trimester and preterm birth. This finding is consistent with the research by Ye et al. (19) and Li et al. (20), suggesting that the second trimester may be an effective exposure window for particulate matter pollution. During this period, exposure to external air pollutants can lead to preterm birth. In the third trimester, PM2.5 exposure significantly increases the probability of preterm birth, which aligns with the findings of Cheng et al. (21). Throughout pregnancy, exposure to PM2.5 and PM10 significantly raises the risk of preterm birth, consistent with the studies by Liu et al. (22), Li et al. (23), and Lamichhane et al. (24). Although the exact mechanism by which PM2.5 and PM10 exposure leads to preterm birth is not entirely clear, some reports suggest that long-term exposure to airborne particulate matter may increase systemic oxidative stress and inflammatory responses in pregnant women, impairing the placenta and causing endocrine disruption, which in turn raises the risk of infection and preterm birth (25). Additionally, research has shown that the association between PM2.5 and PM10 exposure and mtDNA methylation is most significant during early pregnancy, while the association between PM2.5 exposure and mtDNA content is most pronounced in late pregnancy (26). While DNA methylation can occur throughout life, it seems to play a particularly important role in regulating embryonic growth and placental development, which in turn affects birth outcomes (27). Exposure to fine particulate matter may induce systemic oxidative stress, placental dysfunction, and pro-inflammatory responses in pregnant women, potentially leading to inadequate trophoblast invasion or poor placental perfusion, thereby hindering fetal growth and resulting in adverse birth outcomes (28). These biological mechanisms explain how exposure to airborne particulate matter can lead to adverse birth outcomes such as preterm birth, supporting some of the conclusions in this study. However, it is important to consider potential confounding factors, such as maternal health conditions or socioeconomic disparities, as well as differences in pollutant concentrations across regions and variations in air pollution monitoring methods, which may influence the results.

The strength of this study lies in its systematic evaluation of the relationship between exposure to airborne particulate matter (PM2.5, PM10) during different stages of pregnancy (early, mid, late, and entire pregnancy) and preterm birth among a large sample of pregnant women in China, providing scientific evidence for pregnancy interventions. However, there are several limitations: first, the study did not account for the economic status, social background, and nutritional status of the pregnant women, all of which may influence adverse pregnancy outcomes. Additionally, maternal comorbidities, past medical history, and active or passive smoking were not considered, which are all potential confounders. Lastly, air pollutants often occur as mixtures, and it is difficult to analyze the effect of a single pollutant on preterm birth. Furthermore, differences in pollution monitoring methods across studies may lead to variations in pollution concentration measurements, contributing to heterogeneity and affecting the results.

## 5 Conclusion

In conclusion, we found that exposure to airborne particulate matter during the second and third trimesters, as well as throughout pregnancy, is associated with preterm birth. Although the correlation is small and there are potential confounding factors that cannot be fully addressed, this study highlights the risks of prenatal exposure to air pollution and provides some reference points for future research. For example, future studies could collect baseline data from pregnant women with similar cultural, economic, and social backgrounds and analyze the correlation under the same pollution intensity. This could also provide further clues for investigating the biological mechanisms involved.

## Data Availability

The original contributions presented in the study are included in the article/supplementary material, further inquiries can be directed to the corresponding author.
